# Acclimation of the Grapevine *Vitis vinifera* L. cv. Assyrtiko to Water Deficit: Coordination of Structural and Functional Leaf Traits and the Dynamic of Calcium Oxalate Crystals

**DOI:** 10.3390/plants12233992

**Published:** 2023-11-27

**Authors:** Foteini Kolyva, Dimosthenis Nikolopoulos, Panagiota Bresta, Georgios Liakopoulos, George Karabourniotis, Sophia Rhizopoulou

**Affiliations:** 1 Section of Botany, Department of Biology, National and Kapodistrian University of Athens, 15784 Athens, Greece; fotinikoliva@biol.uoa.gr; 2 Laboratory of Plant Physiology and Morphology, Faculty of Crop Science, Agricultural University of Athens, 11855 Athens, Greece; d.nikolopoulos@aua.gr (D.N.); liak@aua.gr (G.L.); 3 Laboratory of Electron Microscopy, Department of Crop Science, Agricultural University of Athens, Iera Odos 75, Votanikos, 11855 Athens, Greece; brestapan@aua.gr

**Keywords:** alarm photosynthesis, chlorophyll fluorescence, grapevine, leaf gas exchange, water potential, water deficit

## Abstract

Grapevine leaves contain abundant CaO_x_ crystals located either within the mesophyll in the form of raphides, or in the bundle sheaths as druses. CaO_x_ crystals function as internal carbon pools providing CO_2_ for a baseline level of photosynthesis, named “alarm photosynthesis”, despite closed stomata; thus, preventing the photoinhibition and the oxidative risk due to carbon starvation under adverse conditions. Structural and functional leaf traits of acclimated grapevine plants (*Vitis vinifera* L. cv. Assyrtiko) were investigated in response to water availability, in order to evaluate the dynamic functionality of CaO_x_. Leaf water potential, leaf area, leaf mass per area, stomatal properties, gas exchange parameters and performance index (PI) were decreased in leaves of vines acclimated to water deficit in comparison to the leaves of well-irrigated vines, although the chlorophyll fluorescence parameters showed that the operational efficiency of the photosystem II (PSII) photochemistry (F_v_/F_m_) did not change, indicating that the photosynthetic apparatus was not subjected to water stress. During the afternoon, more than half of the morning’s existing druses disappeared in the drought-acclimated leaves. Also, the raphides’ area of the drought-acclimated leaves was reduced more than that of the well-watered leaves. The substantial decomposition of druses under water deficit conditions compared to that of the raphides may have important implications for the maintenance of their different though overlapping roles. According to the results, it seems likely that, under water deficit conditions, a mechanism of “alarm photosynthesis” provides an additional tolerance trait in the leaves of *Vitis vinifera* cv. Assyrtiko; hence, leaf structure relates to function.

## 1. Introduction

According to the Intergovernmental Panel on Climate Change (IPCC), the frequency and intensity of episodic extreme weather and climate events, such as heat waves, have increased as a consequence of global warming and will continue to increase even under the medium emission scenarios. It is expected that the frequency, intensity and duration of heatwaves and drought will increase worldwide, especially in some regions which are already drought-prone, predominantly in the Southern Mediterranean, Europe, Southern Amazon and Southern Africa [1]. Additionally, the above-mentioned regions will warm faster than the global average warming rate, which will result in local increases in temperature in dryland areas [2]. According to the RCP8.5 scenario, the region of the Mediterranean Basin is expected to receive approximately 20% less precipitation at the end of the 21st century and this will be a threat for drought-sensitive crops, native and wild plant species. Water deficit is considered to be one of the most common and hazardous environmental stress factors, which negatively affects plant growth and survival. Under drought conditions, plant development depends on their adaptive characteristics and acclimation ability. In considering that increased irrigation is not always a sustainable solution, research linked to drought tolerance mechanisms is critical for understanding the future consequences of climate change on plant survival and crop productivity [3].

*Vitis vinifera* L. is a winter deciduous perennial, long-lived plant of great economic value, grown in regions with Mediterranean climate [4,5,6], as well as in a wider range of climates, such as in semi-arid and tropical regions [5,7]. Summer water deficit is considered the main environmental constraint for plant growth in Mediterranean-type ecosystems. Under Mediterranean climatic conditions, the physiological regulation of water use in response to soil water depletion is essential for species survival, productivity and distribution. Although genotype-dependent drought tolerance has been observed [8,9,10], grapevines are generally adapted to water deficit stress conditions by exhibiting suitable functional and/or structural traits and a pronounced control of water loss by stomatal regulation mediated by ABA [8,11,12,13,14]. Moreover, the photosynthetic mechanism appears to be rather tolerant to mild water shortage [3,8,15,16].

Concerning the water requirement of *V. vinifera* plants grown in the field, it is likely that their performance and life cycle are mainly sustained by rainfall; however, in several regions in Mediterranean countries, irrigation is more and more adopted as a current practice [17,18,19]. The impact of climatic change on vineyards and viticulture is expected to be severe in the Mediterranean region, due to a combination of environmental parameters, such as drought, elevated air temperature and high evaporative demand during the dry period, coupled with the progressive decline in water sources [14,20,21,22,23]; the last impact will limit the application of irrigation as a last future shelter [22]. The migration of vineyards to higher elevations and/or new, more suitable regions and the adoption of new tolerant varieties have been suggested as possible consequences of climate change, as well as possible adaptation strategies [24,25,26].

Plants’ drought tolerance mechanisms include functional traits combined with structural traits aiming at maximum water use efficiency [27,28]. A mechanism of similar targeting was found in the form of a biochemical appendance and was named “alarm photosynthesis” [29]; this acts as a diurnally two-phase biochemical pathway by collecting and saving carbon in the form of calcium oxalate (CaO_x_) crystals during the night and by providing subsidiary CO_2_ for photosynthetic assimilation during the day. In fact, CO_2_ is derived from the degradation of these crystals and the subsequent breakdown of oxalate [29,30]. It seems likely that the function of this biochemical appendance is induced by ABA, when stomata are closed under water deficit conditions [31]. Thus, in “alarm photosynthesis”, CaO_x_ crystals can be used as internal carbon pools, irrespectively of the availability of the atmospheric CO_2_, preventing the plant’s water loss [32].

Grapevine leaves contain abundant CaO_x_ crystals in the form of raphide bundles in crystal idioblasts within the mesophyll [33,34,35,36,37]. Raphides are surrounded by an organic matrix consisting of two structural phases, i.e., membrane chambers enclosing crystals and a water-soluble phase that contains polysaccharides and glycoproteins [38,39]. Moreover, additional CaO_x_ crystals in the form of druses are located in the bundle sheaths. The formation of crystals takes place at the very early leaf developmental stages and the maximum density has been observed in very young, expanding leaves [36,37,38,39]. During leaf development, the properties of the idioblastic cells (i.e., crystal number, size and density) change in a coordinated way with leaf area and it seems likely that each idioblast is connected to and “serves” a finite number of adjacent cells. Moreover, the size of the crystals is also related to aspects of gas exchange rates [40,41].

Our knowledge on grapevine drought stress physiology has significantly increased [42,43,44], but short and middle-term acclimation responses of non-irrigated field-grown grapevines have rarely been studied [45,46,47,48,49,50]. The lack of a mechanistic understanding of many drought responses remains a significant unknown issue linked to the accuracy of modeling future climatic scenarios. In order to increase the efficiency of management practices or the resiliency of vineyards and create drought-tolerant varieties and rootstocks, a better understanding of *V. vinifera’s* responses to water shortage is required.

The objective of the present work was to evaluate the dynamics of CaO_x_ crystals in *Vitis vinifera* cv. Assyrtiko grown under water deficit conditions. *Vitis vinifera* cv. Assyrtiko is a vine with white grapes, indigenous on the island of Santorini (36.3932° N, 25.4615° E) and widely planted throughout Greece and on the Cyclades’ islands [51]; also, it is being grown in Australia and California. It is noteworthy that own-rooted and phylloxera-free *Vitis vinifera* cv. Assyrtiko has been cultivated on the volcanic dry soil of Santorini for thousands of years [52]. Our hypothesis was linked to the effect of water deficit on vines, which is followed by stomatal closure and causes the decomposition of both types of CaO_x_ crystals (i.e., druses and raphides) in the leaves, providing internal CO_2_ for a baseline level of photosynthesis. It is expected that the obtained results will help us to understand how the mechanism of an “alarm photosynthesis” may enhance the drought tolerance of *V. vinifera* cv. Assyrtiko.

## 2. Results

### 2.1. Leaf Functional Parameters

The leaf area and leaf mass per area, as well as the stomatal length, were significantly higher (*p* < 0.05) in well-watered plants, in comparison with the grapevines exposed to water deficit (Table 1); meanwhile, the stomatal density, needed for photosynthesis to occur, was comparable between the well-watered and water-stressed grapevines (Table 1).

**Figure 1:**
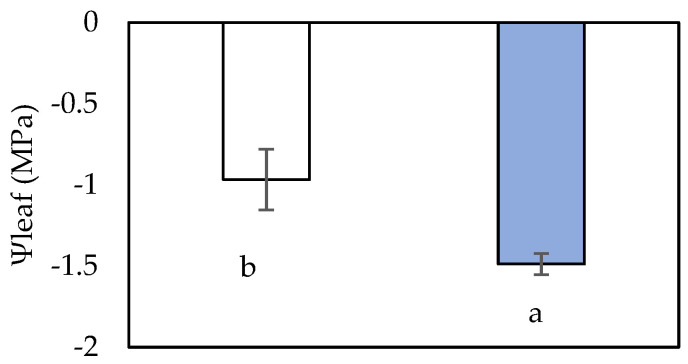
Leaf water potential (Ψ_leaf_) of well-watered (blue bar) and water-deficit-stressed (white bar) *Vitis vinifera* cv. Assyrtiko plants. The values are means of seven replicates ± S.E. Significant differences (*p* < 0.05) of mean values are marked using lowercase letters.

The imposed middle-term water deficit treatment in grapevines caused a significant decline in midday leaf water potential (Ψ_leaf_) (Figure 1), which is also indicative of the failing soil moisture availability. When water became less available, the maximum photosynthetic capacity (A_max_), transpiration rate (E), stomatal conductance (g_s_), water use efficiency (WUE) and intrinsic water use efficiency (WUE_i_) declined in leaves of plants subjected to water-deficit treatment in comparison to the leaves of well-watered plants (Table 2). In addition, the stomatal length, which affects the stomatal aperture, seems to influence the gas exchange parameter under declining leaf water potentials.

The chlorophyll fluorescence parameters (F_v_/F_m_) showed that the operational efficiency of the photosystem II (PSII) in the water deficit-acclimated leaves was substantially sustained (Table 2). PI_total_, which indicates how effectively the photosynthetic system converts light energy into chemical energy, was lower in leaves subjected to water deficit conditions.

### 2.2. Leaf Structural Modifications

**Figure 2:**
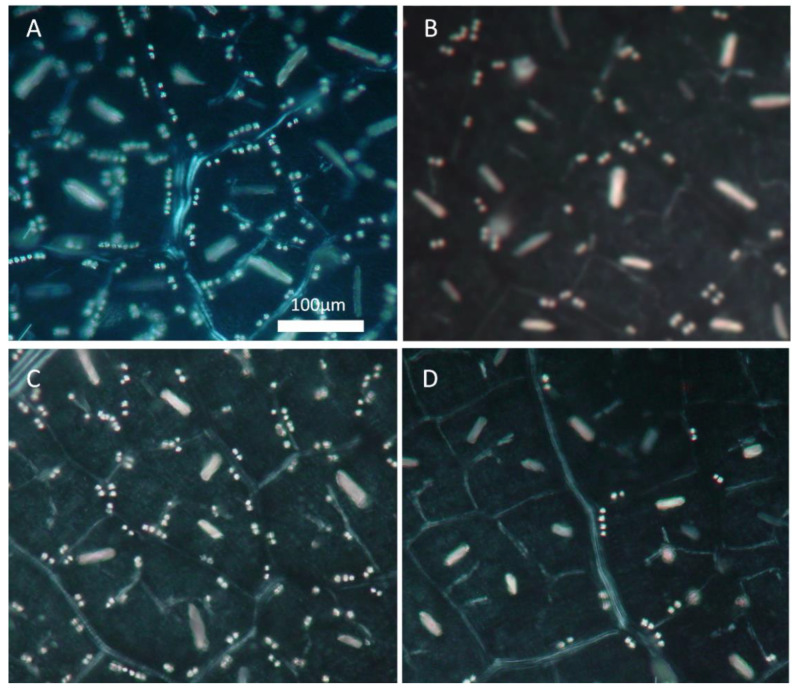
Representative, paradermal view of raphide and druse idioblasts of mature *Vitis vinifera* cv. Assyrtiko leaves under polarized light. (**A**) Morning well-watered sample. (**B**) Afternoon well-watered sample. (**C**) Morning water-deficit-stressed sample. (**D**) Afternoon water-deficit-stressed sample. (Scale bar: 100 μm).

The distribution and the dimensions of the CaO_x_ crystals were measured in the leaves of the control and water-deficit-stressed plants during early morning and late afternoon hours, when the maximum difference in the size of these inclusions is expected. The density and the width of the raphides did not vary between treatments and sampling time; however, in the early morning sampling, drought-acclimated leaves possessed larger and longer raphides, exhibiting a higher area, than the control leaves (Table 3). This indicates a larger carbon source, which serves the alarm photosynthesis of the drought-acclimated leaves. In the afternoon sampling, a significantly lower area of raphides was recorded in both treatments, compared to the area of the early morning sampling. Thus, drought-acclimated leaves showed larger differences between the morning and afternoon samplings compared to the control leaves, indicating a much more active alarm photosynthesis under stress conditions. In the case of druses, the estimation of their dimensions was practically inapplicable; nevertheless, changes were all-out: more than half of the morning existing druses disappeared during the afternoon sampling in the drought-acclimated leaves (Figure 2C,D). This caused a significant reduction in the density of the druses in the afternoon sampling (Table 3). The change in the density of druses of the control leaves between the morning and afternoon samplings, although significant, was clearly of lower magnitude compared to those of the drought-acclimated leaves (Table 3).

## 3. Discussion

The leaves of *Vitis vinifera* cv. Assyrtiko, which were expanded after the onset of the imposed drought conditions, showed an altered phenotype due to a plethora of structural and functional modifications. The water deficit caused a significant reduction in the leaf area and leaf mass per area (Table 1), which is in agreement with previous findings [53,54,55,56,57]. The drought-acclimated, smaller in size leaves showed a slightly higher stomatal density and reduced stomatal length compared to those of the leaves of the well-watered plants (Table 1); this may be consistent with a stomatal response to the pool size of a carbon fixing substrate [58].

The results linked to transpiration rate (E), stomatal conductance (g_s_), maximum photosynthetic capacity (A_max_), intrinsic water use efficiency (WUE_i_) and water use efficiency (WUE) are in accordance with previous investigations [9,49,59,60]. Concerning the water use efficiency, there are conflicting results in the literature, i.e., a reduced water supply to grapevines may result in either decreased or increased WUE_i_ [61,62,63]. However, in our study, the decline in WUE_i_ was due to a significant decline in A_max_, suggesting that the leaves exhibited either high photorespiration rates or biochemical compromise [49].

It has been shown that CO_2_ derived from oxalate through crystal decomposition can sustain a minimum rate of photosynthesis and abstains stress in the photosynthetic apparatus [29]. In contrast, the performance index (PI_total_)—which is a very sensitive parameter providing quantitative information about the overall condition and vitality of the plant, and which is usually correlated with the net assimilation rate (A) [64,65,66]—declined in leaves exposed to water deficit conditions (Table 2).

It has been argued that CaO_x_ crystals represent a dynamic system, which shows a gradual decomposition during the day and a gradual recovery during the night [29,40]. The occurrence of CaO_x_ crystals at the interspecific level has been described as a trait linked to drought tolerance and very important for xerophytes and desert plants [30]. Also, the presence of oxalate crystals has been represented as a taxonomic trait [67].

The results of the present study confirm that, at the intraspecific level, there is a connection between drought conditions, imposed via different watering regimes, and the decomposition of CaO_x_ crystals, which has been observed in a number of taxonomically different plant species [29,59,67,68]. The water deficit is a very important issue for plant survival not only due to tissue and organ dehydration, but also due to carbon deficit within the mesophyll during stomatal closure [69,70,71]. Under these conditions, CaO_x_ crystals function as carbon pools, providing CO_2_ for alarm photosynthesis through oxalate breakdown [29]; hence, preventing the negative carbon balance, which can lead to an exhaustion of carbon reserves, as well as to an imbalance between incident energy and available intercellular CO_2_ and thus photooxidative risk [29]. The F_v_/F_m_ ratio of the water-deficit-stressed plants was comparable to the well-watered plants; chlorophyll fluorescence measurements showed that the operational efficiency of the photosystem II in the drought-acclimated leaves was sustained despite stomatal closure (Table 2); such mechanism can be seen as an important component of tolerance to drought stress [66,72]. However, the lower performance index (PI_total_) of the leaves subjected to water deficit compared to the well-watered leaves indicates that the activity of alarm photosynthesis (supported by CO_2_ derived from oxalate through crystal decomposition) was lower than the normal C3 photosynthesis (supported by CO_2_ derived from the open stomata).

An interesting finding of the present research was that the decomposition of druses under water deficit conditions was more intense than that of the raphides. Taking into account that carbon atoms in the CaO_x_ crystals are derived from sources other than C3 photosynthesis and that oxalate from the root is transferred to the leaves via the xylem [31], we argue that raphides and druses may play different roles. On one hand, raphides, located between photosynthetic cells in the mesophyll, may provide CO_2_ directly to these cells. On the other hand, druses located in the bundle sheaths may constitute an intermediate oxalate source derived from the root, via a gradient required for such translocation. Further research is required to fully test this hypothesis.

## 4. Material and Methods

### 4.1. Research Sites and Plant Material

**Figure 3:**
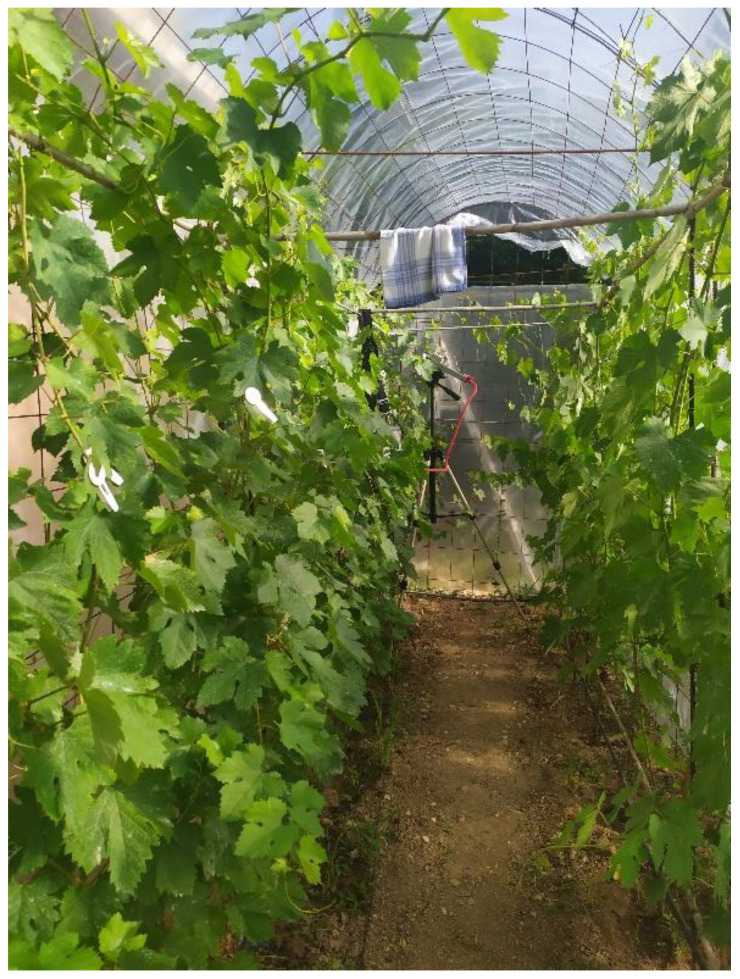
View of the vineyard, on Aegina Island in Greece.

**Figure 4:**
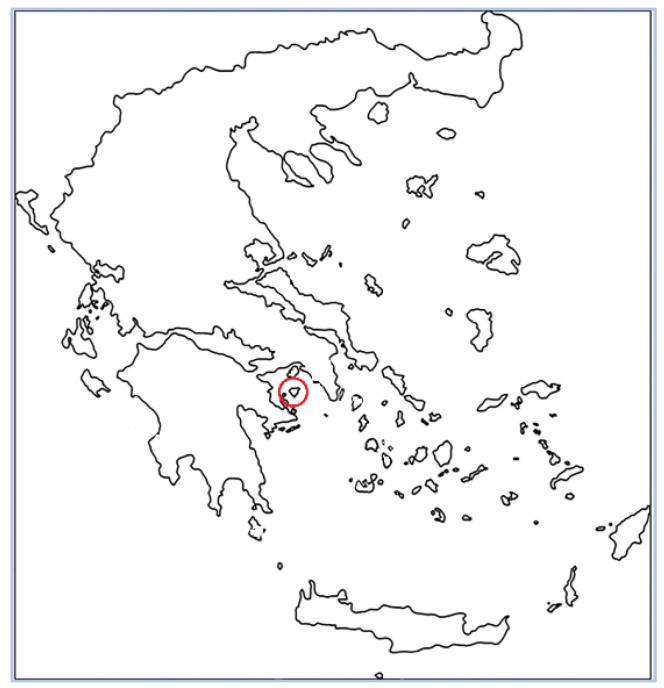
Abstract map of the territory of Greece, where the location of Aegina Island is indicated by the red circle.

**Figure 5:**
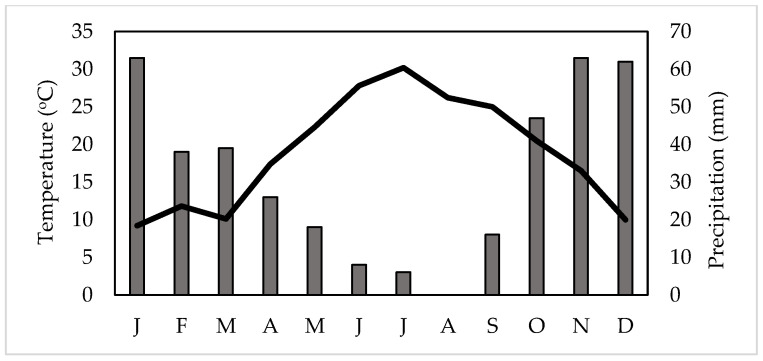
Ombrothermic diagram (Precipitation scale = 2 × Temperature scale) for the study site on Aegina Island for two consecutive years, i.e., 2021 and 2022. Mean monthly precipitation is indicated by dark bars and mean monthly temperature by the black line; the order of months is from January (J) to December (D).

The *Vitis vinifera* cv. Assyrtiko plants were initially grown in in a growth chamber, in 6 L pots, filled with a soil mix composed by 55% sand, 30% clay, 14% silt and 1% organic matter. Then, the plants were transported, transplanted and maintained growing on the ground in a greenhouse (Figure 3) on Aegina Island (37.7477 N, 23.5386 E) (Figure 4), during 2021, i.e., a year prior of the imposed water deficit experimental procedure; there, the soil was composed by 63.2% sand, 19.4% clay and 17.4% silt. The average PPFD (photosynthetic photon flux density), although not stable throughout the daily photoperiod, was approximately 1600 μmol m^–2^ s^–1^, during the gas exchange measurements. The distances between the planting rows were 2.2 m and 1.2 m between vine-plants of the same row [73]. The soil fertilizer was 11-15-15 NPK (nitrogen, phosphorus, potassium, Nutriplus, Aspropyrgos, Greece). Monthly mean temperature and monthly precipitation, during two years in the study site (Figure 5), were obtained from the closest meteorological station provided by the Hellenic National Meteorological Service [74]. Inside the greenhouse, the air temperature was 2–3 °C higher than that of the outside ambient conditions (Figure 5). Prior to the measurements, the predawn relative humidity in the greenhouse varied from 70% to 80% [75]. Also, the mean annual relative humidity in the surrounding ambient environment of the research site has been estimated at 65–75% [76]. Daily, the windows and the door of the greenhouse were manually operated, i.e., they opened at sunrise and closed at sunset; thus, the relative humidity in the greenhouse was reduced during the daytime.

During the second year (2022), the two-year-old grafted vines (*Vitis vinifera* cv. Assyrtiko on rootstock Paulsen 1103P), were divided into two groups and assigned to each of the two watering treatments; there were only two groups of plants, each on one side of the greenhouse. The first group of plants, i.e., half of the plants, were kept under well-watered conditions for the whole experimental period, i.e., they were irrigated using low-volume drip irrigation without causing runoff [77]. The plants were diurnally irrigated at 09:00 a.m., from April to September. In August, when midday temperatures were higher than 30 °C and the mean monthly rainfall was 0 mm (Figure 4), the plants were also irrigated at 20:00 p.m. The second group of plants was exposed to a soil water content 30% lower than that of the first group, from April to September. The well-watered plants were supplied with 10 L of tap water, while the plants subjected to deficit irrigation were irrigated with 7 L of tap water. During the days of the experiments and measurements, water was supplied to the vines, after leaf harvesting. There was not any barrier or membrane buried in the soil. It is noteworthy that it has been known that water flow into the main roots of *Vitis vinifera* plants via the lateral root pathway is likely to be negligible, in comparison to the direct radial flow pathway, as only about 1% of the surface area of the main roots is occupied by lateral roots, leaving the remaining 99% of the main root surface area available for the direct radial flow pathway [78]. Also, the root diameters of *Vitis vinifera* were smaller for water-stressed plants compared to well-watered plants [78].

### 4.2. Morphological and Anatomical Estimations

Leaf trichomes of *Vitis vinifera* cv. Assyrtiko were gently removed by pressing a clear adhesive tape on the leaf surface. Measurements of stomatal density and stomatal length were performed on the abaxial leaf surface of intact fresh leaves; five leaves from different plants of both the well-watered and the droughting treatment were used. The Axiolab microscope (Zeiss, Jena, Germany) and the digital camera DSC-S75 (Sony, Tokyo, Japan) were used for observations and for photographing the anatomical characteristics (magnification 20×). In this method, a small area on the leaf surface was painted by a thick patch of clear nail polish. When the nail polish was completely dry, a piece of clear cellophane tape was used to peel out the nail polish patch by pulling the corner of the tape, as well as the nail polish along with the leaf peel. Then, leaf impressions were examined and photographed under a light microscope [79]. The images were analyzed using Image-Pro Plus (v.5.1.0.20) [80].

The leaf area (LA) was investigated using leaf photographs by image analysis (Image-Pro Plus, v.5.1.0.20). Leaf samples were oven-dried at 70 °C for 48 h and the leaf dry weight was measured in an analytical balance. Leaf mass per area (LMA) was estimated as the ratio of leaf dry matter versus leaf area (g m^−2^). Each value is the mean of five replicates.

### 4.3. Leaf Water Status

The midday leaf water potential (Ψ_leaf_) of well-irrigated and water-deficit-stressed grapevines *Vitis vinifera* cv. Assyrtiko [81] was measured in seven mature leaves, from different plants, adjacent and/or in proximity to the leaves used for the gas exchange measurements, using a Skye Scholander-type pressure chamber (Skye Instruments Ltd., Wales, UK). The mature leaves were inserted into the pressure chamber where the pressure was raised slowly at a constant rate (~0.01 MPa s^−1^), and the pressure was recorded when a water meniscus started to be formed on the cut petiole (midvein of leaf) surface. Each value is the mean of seven replicates.

### 4.4. Chlorophyll Fluorescence

Chlorophyll fluorescence measurements were performed on fully expanded leaves, during morning hours (i.e., between 07:15 a.m. and 10:50 a.m.). The in vivo chlorophyll fluorescence parameters were recorded using continuous fluorescence induction fluorometry in order to study the fast fluorescence transient (OJIP parameters), using a portable chlorophyll fluorometer (Handy PEA, Hansatech Instruments, King’s Lynn, UK). Five leaves from different plants exposed to the well-watered and the droughting treatment were used. Each leaf was acclimated for 30 min, prior to the measurements, using dark leaf clips. A bank of three red LEDs (peak at 650 nm) providing 3000 μmol (photon) m^−2^ s^−1^ was used for excitation. Fluorescence was recorded from 10 μs to 2 s with intervals of 10 μs, 100 μs, 1 ms, 10 ms and 100 ms between the readings, for periods of 10–300 μs, 0.3–3 ms, 3–30 ms, 30–300 ms and 0.3–2 s, respectively. Cardinal points used for further calculation of biophysical parameters were as follows: maximal fluorescence intensity (F_m_, when all reaction centers (RCs) are closed), minimum fluorescence intensity (F_0_, when all RCs are open) and fluorescence intensity at 2 and 30 ms, at the J and I steps, respectively (F_J_ and F_I_), and at 300 μs (F_300μs_). Relative variable fluorescence at J [V_J_ = (F_J_ − F_0_)/(F_m_ − F_0_)] and I [V_I_ = (F_I_ − F_0_)/(F_m_ − F_0_)] steps, and at 300 μs [V_K_ = (F_K_ − F_o_)/(F_m_ − F_0_)] was also used. Fluorescence data were then transformed in a logarithmic time scale and the derived polyphasic curve was analyzed according to JIP-test [82], as extended to analyze events around PSI [83,84].

### 4.5. Leaf Gas Exchange Parameters

Measurements of gas exchange parameters were performed on fully expanded leaves using a portable open-circuit gas-exchange instrument (LCPro+, ADC BioScientific Ltd., Hoddesdon, UK), equipped with a broad leaf chamber enclosing 6.25 cm^2^ of leaf area. Five leaves from different well-watered and water-stressed plants were used. Temperature and relative air humidity inside the chamber were 29.3 ± 2.1 °C, and 41.3 ± 3.6%, respectively. Gas exchange parameters (A: net rate of CO_2_ assimilation, E: transpiration rate and g_s_: stomatal conductance) were measured using light curves at ambient CO_2_ atmospheric concentration (408 ± 8 μmol CO_2_ mol^−1^ air), from 07:15 a.m. to 10:50 a.m. The plants were exposed to a gradient of photosynthetic photon flux density (PPFD), ranging from 0 μmol m^−2^ s^−1^ to 1840 μmol quanta m^−2^ s^−1^, following acclimation for 3 min at each intensity (three readings at steady-state conditions were recorded per replicate and per light level). The water use efficiency (WUE) is the ratio of carbon assimilated by the plant (A) versus the amount of water lost via transpiration (E) and was calculated as instantaneous WUE = A/E and intrinsic WUE_i_ = A/g_s_.

### 4.6. Properties of Calcium Oxalate Crystals

The number, the density and the area of CaO_x_ crystals (raphides and druses) were measured on leaves bleached with sodium hypochlorite solution 2.5 % (*w*/*v*) for 48 h, in order to enhance the visibility. In the case of *V. vinifera*, the birefringence properties of the crystals make them more visible; thus, the leaves were viewed with a polarizing filter to further improve measurement accuracy. Seven images per leaf, of five leaves from different well-irrigated and water-stressed plants, during morning (07:00) and afternoon (19:00) random sampling, were acquired with an Axiolab microscope (Zeiss, Jena, Germany), digital camera DSC-S75 (Sony, Tokyo, Japan) and a polarizing filter. All crystal dimensions shown in each image were obtained by digital image analysis (Image Pro Plus, v. 5.1.0.20). Concerning the measurements of dimensions of the inclusions, the raphides were treated as cylinders. Also, empty raphides, total number of raphides, length and width of raphides were measured. In addition, the density of druses was investigated. Each value is the mean of seven replicates. The volume of the raphides (V) was calculated according to the equation V = π·r^2^·h, where r = ½ of width and h: length; therefore, in this case, standard error values are not included in Table 3.

### 4.7. Statistical Analysis

Grubbs’ test or ESD method (extreme studentized deviate) was performed, to determine whether the most extreme value is a significant outlier. The outlier value was removed and replaced by the mean value. Statistical tests were performed using past statistical program v. 4.13 (University of Oslo, Oslo, Norway). For comparison of means after normality and equal variance criteria between sampling hours (morning—afternoon) and treatments (control—drought) linked to the distribution and properties of the inclusions, two-way ANOVA was used, for main effect analysis, followed by Tukey–Kramer (LSD) test. For comparing the means between the treatments, an analysis of t-Student’s or Kruskal–Wallis tests was performed on the presented parameters at *p* < 0.05.

## 5. Conclusions

The acclimation of grapevine leaves to water deficit conditions involves constraints on their area and a reduction in gas exchange and carbon reserve in the form of CaO_x_ crystals; the substantial decomposition of these crystals during the day indicates active “alarm photosynthesis”. From the findings of the present study, it seems likely that the involvement of CaO_x_ crystals is a dynamic system that contributes to the drought tolerance of *Vitis vinifera* cv. Assyrtiko. Moreover, both crystal types (raphides and druses) may coordinate in order to prevent carbon starvation and oxidative risk, under water-deficit stress conditions, due to stomatal closure. Given the rapid leaf growth of *Vitis vinifera* cv. Assyrtiko in spring and leaf exposure to the dry summer conditions in the eastern Mediterranean, these structural and functional traits (before growth cessation) provide valuable information linked to adaptive process and yield, under the considered ambient conditions.

## Figures and Tables

**Table 1:** Morphological and anatomical characteristics of the leaves of *Vitis vinifera* cv. Assyrtiko plants grown under water deficiency for five months. Values of the stomatal density and stomatal length are means of seven replicates ± S.E. Values of leaf area and leaf mass per area (LMA) are means of five replicates ± S.E. Significant differences of mean values according to LSD test are marked using lowercase superscript letters that are given separately on each line; means with the same letter are not significantly different (*p* < 0.05).

Parameter (Units)	Well-Watered	Water-Deficit-Stressed
Stomatal density (mm^−2^)	25.91 ± 2.80 ^a^	27.34 ± 3.20 ^a^
Stomatal length (μm)	55.10 ± 3.10 ^a^	42.40 ± 1.80 ^b^
Leaf area (cm^2^)	140.84 ± 14.53 ^a^	108.75 ± 8.90 ^b^
LMA (g m^−2^)	42.40 ± 1.66 ^a^	39.26 ± 2.52 ^b^

**Table 2:** Transpiration rate (E), stomatal conductance (g_s_), maximum photosynthetic capacity (A_max_), intrinsic water use efficiency (WUE_i_), water use efficiency (WUE), chlorophyll fluorescence parameter F_v_/F_m_ and performance index of the leaves of *Vitis vinifera* cv. Assyrtiko grown under water deficiency. Significant differences of mean values according to LSD test are marked using lowercase superscript letters, which are given separately on each line, according to each variable. Values are means of five replicates ± S.E.

Parameter (Units)	Well-Watered	Water-Deficit-Stressed
E (mmol H_2_O m^−2^ s^−1^)	2.87 ± 0.08 ^a^	1.99 ± 0.09 ^b^
g_s_ (mol H_2_O m^−2^ s^−1^)	0.15 ± 0.01 ^a^	0.09 ± 0.00 ^b^
A_max_ (μmol CO_2_ m^−2^ s^−1^)	15.12 ± 0.92 ^a^	4.82 ± 0.52 ^b^
WUE_i_ (μmol CO_2_ mmol^−1^ H_2_O)	99.52 ± 4.33 ^a^	53.36 ± 6.26 ^b^
WUE (μmol CO_2_ mol^−1^ H_2_O)	5.23 ± 0.24 ^a^	2.44 ± 0.31 ^b^
F_v_/F_m_	0.80 ± 0.01 ^a^	0.79 ± 0.01 ^a^
PI_total_	3.61 ± 0.54 ^a^	2.58 ± 0.19 ^b^

**Table 3:** Mean values of traits of raphides and druses of the leaves of *Vitis vinifera* cv. Assyrtiko grown under water deficiency. Values are means of seven replicates ± standard error. Significant differences (*p <* 0.05) of mean values according to LSD test are marked using lowercase superscript letters that are given separately on each line according to each variable.

Parameter (Units)	Well-Watered: Morning	Water-Deficit-Stressed: Morning	Well-Watered: Afternoon	Water-Deficit-Stressed: Afternoon
Raphides’ Length (μm)	73.4 ± 4.91 ^ab^	84.0 ± 3.10 ^a^	69.0 ± 2.24 ^b^	66.0 ± 1.34 ^b^
Raphides’ Width (μm)	24.4 ± 0.20 ^a^	22.9 ± 0.59 ^a^	23.8 ± 0.63 ^a^	22.7 ± 0.36 ^a^
Raphides’ Area (μm^2^)	6449.4 ± 424.90 ^b^	7203.9 ± 312.74 ^a^	5836.2 ± 269.95 ^c^	5545.6 ± 163.62 ^c^
Raphides’ Volume (μm^3^)	34,304.05 ^a^	34,579.60 ^a^	30,681.22 ^b^	26,697.17 ^c^
Raphides’ Density (mm^–2^)	13.14 ± 0.57 ^a^	13.17 ± 0.70 ^a^	12.55 ± 1.24 ^a^	12.90 ± 0.60 ^a^
Druses’ Density (mm^–2^)	108.09 ± 10.92 ^a^	82.30 ± 10.37 ^c^	97.49 ± 11.77 ^b^	40.61 ± 5.96 ^d^

## Data Availability

The data are available from the authors upon request.

## References

[1] Jia G., Shevliakova E., Artaxo P., De Noblet-Ducoudré N., Houghton R., House J., Kitajima K., Lennard C., Popp A., Sirin A. (2019). Land–climate interactions. Special Report on Climate Change and Land: An IPCC Special Report on Climate Change, Desertification, Land Degradation, Sustainable Land Management, Food Security, and Greenhouse Gas Fluxes in Terrestrial Ecosystems.

[2] Schlaepfer D.R., Bradford J.B., Lauenroth W.K., Munson S.M., Tietjen B., Hall S.A., Wilson S.D., Duniway M.C., Jia G., Pyke D.A. (2017). Climate change reduces extent of temperate drylands and intensifies drought in deep soils. Nat. Commun..

[3] Gambetta G.A., Herrera J.C., Dayer S., Feng Q., Hochberg U., Castellarin S.D. (2020). The physiology of drought stress in grapevine: Towards an integrative definition of drought tolerance. J. Exp. Bot..

[4] Renfrew J.M. (2003). The Origins and Ancient History of Wine.

[5] Savo V., Kumbaric A., Caneva G. (2016). Grapevine (*Vitis vinifera* L.) symbolism in the ancient Euro-Mediterranean cultures. Econ. Bot..

[6] Moutinho-Pereira J., Gonçalves B., Bacelar E., Cunha J.B., Countinho J., Correira C.M. (2015). Effects of elevated CO_2_ on grapevine (*Vitis vinifera* L.): Physiological and yield attributes. Vitis-J. Grapevine Res..

[7] Leão P.C.S. (2018). Genetic resources for table-grape breeding in Brazilian tropical semi-arid regions. Acta Hortic..

[8] de Oliveira J.B., Egipto R., Laureano O., de Castro R., Pereira G.E., Ricardo-da-Silva J.M. (2019). Chemical characteristics of grapes cv. Syrah (*Vitis vinifera* L.) grown in the tropical semiarid region of Brazil (Pernambuco state): Influence of rootstock and harvest season. J. Sci. Food Agric..

[9] Chaves M.M., Zarrouk O., Francisco R., Costa J.M., Santos T., Regalado A.P., Rodrigues M.L., Lopes C.M. (2010). Grapevine under deficit irrigation: Hints from physiological and molecular data. Ann. Bot..

[10] Schultz H.R., Stoll M. (2010). Some critical issues in environmental physiology of grapevines: Future challenges and current limitations. Aust. J. Grape Wine Res..

[11] Shelden M.C., Vandeleur R., Kaiser B.N., Tyerman S.D. (2017). A comparison of petiole hydraulics and aquaporin expression in an anisohydric and isohydric cultivar of grapevine in response to water-stress induced cavitation. Front. Plant Sci..

[12] Liakopoulos G., Nikolopoulos D., Karabourniotis G. (2007). The first step from light to wine: Photosynthetic performance and photoprotection of grapevine (*Vitis vinifera* L.) leaves. Funct. Plant Sci. Biotechol..

[13] Lovisolo C., Lavoie-Lamoureux A., Tramontini S., Ferrandino A. (2016). Grapevine adaptations to water stress: New perspectives about soil/plant interactions. Theor. Exp. Plant Physiol..

[14] Marusig D., Tombesi S. (2020). Abscisic acid mediates drought and salt stress responses in *Vitis vinifera*—A Review. Int. J. Mol. Sci..

[15] MacMillan P., Teixeira G., Lopes C.M., Monteiro A. (2021). The role of grapevine leaf morphoanatomical traits in determining capacity for coping with abiotic stresses: A review. Ci. Técn. Vit..

[16] Flexas J., Escalona J.M., Medrano H. (1998). Down-regulation of photosynthesis by drought under field conditions in grapevine leaves. Aust. J. Plant Physiol..

[17] Dayer S., Reingwirtz I., McElrone A.J., Gambetta G.A., Cantu D., Walker M.A. (2019). Response and recovery of grapevine to water deficit: From genes to physiology. The Grape Genome.

[18] Cifre J., Bota J., Escalona J.M., Medrano H., Flexas J. (2005). Physiological tools for irrigation scheduling in grapevine (*Vitis vinifera* L.): An open gate to improve water-use efficiency?. Agric. Ecosyst. Environ..

[19] Cancela J.J., Trigo-Córdoba E., Martínez E.M., Rey B.J., Bouzas-Cid Y., Fandiño M., Mirás-Avalos J.M. (2016). Effects of climate variability on irrigation scheduling in white varieties of *Vitis vinifera* (L.) of NW Spain. Agric. Water Manag..

[20] Canoura C., Kelly M.T., Ojeda H. (2018). Effect of irrigation and timing and type of nitrogen application on the biochemical composition of *Vitis vinifera* L. cv. Chardonnay and Syrah grapeberries. Food Chem..

[21] van Leeuwen C., Destrac-Irvine A., Dubernet M., Duchêne E., Gowdy M., Marguerit E., Pieri P., Parker A., de Rességuier L., Ollat N. (2019). An update on the impact of climate change in viticulture and potential adaptations. Agronomy.

[22] Fraga H. (2020). Climate Change: A new challenge for the winemaking sector. Agronomy.

[23] Santos J.A., Fraga H., Malheiro A.C., Moutinho-Pereira J., Dinis L.-T., Correia C., Moriondo M., Leolini L., DiBari C., Costafreda-Aumedes S. (2020). A review of the potential climate change impacts and adaptation options for European viticulture. Appl. Sci..

[24] Arias L.A., Berli F., Fontana A., Bottini R., Piccoli P. (2022). Climate change effects on grapevine physiology and biochemistry: Benefits and challenges of high altitude as an adaptation strategy. Front. Plant Sci..

[25] Mosedale J.R., Abernethy K.E., Smart R.E., Wilson R.J., Maclean I.M.D. (2016). Climate change impact and adaptive strategies: Lessons from the grapevine. Glob. Chang. Biol..

[26] De Pascale A., Giannetto C., Zirilli A., Alibrandi A., Lanfranchi M. (2023). How Mediterranean winegrowers perceive climate change. AIMS Agric. Food.

[27] Meletiou-Christou M.S., Rhizopoulou S. (2012). Constraints of photosynthetic performance and water status of four evergreen species co-occurring under field conditions. Bot. Stud..

[28] Meletiou-Christou M.S., Rhizopoulou S. (2017). Leaf functional traits of four evergreen species growing in Mediterranean environmental conditions. Acta Physiol. Plant..

[29] Tooulakou G., Giannopoulos A., Nikolopoulos D., Bresta P., Dotsika E., Orkoula M.G., Kontoyannis C.G., Fasseas C., Liakopoulos G., Klapa M. (2016). “Alarm Photosynthesis”: Calcium oxalate crystals as an internal CO_2_ source in plants. Plant Physiol..

[30] Karabourniotis G., Horner H.T., Bresta P., Nikolopoulos D., Liakopoulos G. (2020). New insights on the functions of carbon-calcium-inclusions in plants. New Phytol..

[31] Tooulakou G., Giannopoulos A., Nikolopoulos D., Bresta P., Dotsika E., Orkoula M.G., Kontoyannis C.G., Fasseas C., Liakopoulos G., Klapa M.I. (2016). Reevaluation of the plant “gemstones”: Calcium oxalate crystals sustain photosynthesis under drought conditions. Plant Sign. Behav..

[32] Tooulakou G., Nikolopoulos D., Dotsika E., Orkoula M.G., Kontoyiannis C.G., Liakopoulos G., Klapa M.I., Karabourniotis G. (2018). Changes in size and composition of pigweed (*Amaranthus hybridus* L.) calcium oxalate crystals under CO_2_ starvation conditions. Physiol. Plant..

[33] Fabbri A., Benelli C., di Collalto G. (1992). Calcium oxalate crystals in vegetative and reproductive organs of the grapevine. Acta Hortic..

[34] Arnott H.J., Webb M.A. (2000). Twinned raphides of calcium oxalate in grape (*Vitis*): Implications for crystal stability and function. Int. J. Plant Sci..

[35] Jauregui-Zuniga D., Reyes-Grajeda J.P., Sepulveda-Sanchez J.D., Whitaker J.R., Moreno A. (2003). Crystallochemical characterization of calcium oxalate crystals isolated from seed coats of *Phaseolus vulgaris* and leaves of *Vitis vinifera*. J. Plant Physiol..

[36] De Bolt S., Hardie J., Tyerman S., Ford C.M. (2004). Composition and synthesis of raphid crystals and druse crystals in berries of *Vitis vinifera* L. cv. Cabernet Sauvignon: Ascorbic acid as precursor for both oxalic and tartaric acids as revealed by radiolabelling studies. Austr. J. Grape Wine Res..

[37] Webb M.A., Suga S., Nakahara H. (1991). Analysis of the organic matrix associated with calcium oxalate crystals in *Vitis mustangensis*. Mechanisms and Phylogeny of Mineralization in Biological Systems.

[38] Webb M.A., Cavaletto J.M., Carpita N.C., Lopez L.E., Arnott H.J. (1995). The intravacuolar organic matrix associated with calcium oxalate crystals in leaves of *Vitis*. Plant J..

[39] Horner H.T., Wagner B.L., Khan S.R. (2020). Calcium oxalate formation in higher plants. Calcium Oxalate in Biological Systems.

[40] Giannopoulos A., Bresta P., Nikolopoulos D., Liakopoulos G., Fasseas C., Karabourniotis G. (2019). Changes in the properties of calcium-carbon inclusions during leaf development and their possible relationship with leaf functional maturation in three inclusion-bearing species. Protoplasma.

[41] Karabourniotis G., Liakopoulos G., Bresta P., Nikolopoulos D. (2021). The optical properties of leaf structural elements and their contribution to photosynthetic performance and photoprotection. Plants.

[42] Lukšić K., Mucalo A., Smolko A., Brkljačić L., Marinov L., Hančević K., Ozretić Zoković M., Bubola M., Maletić E., Karoglan Kontić J. (2023). Biochemical Response and Gene Expression to Water Deficit of Croatian Grapevine Cultivars (*Vitis vinifera* L.) and a Specimen of *Vitis sylvestris*. Plants.

[43] Marta A.E., Slabu C., Covasa M., Motrescu I., Lungoci C., Jitareanu C.D. (2023). Influence of Environmental Factors on Some Biochemical and Physiological Indicators in Grapevine from Copou Vineyard, Iasi, Romania. Agronomy.

[44] Frioni T., Pastore C., Diago M.P. (2023). Resilience of grapevine to climate change: From plant physiology to adaptation strategies-Volume II. Front. Plant Sci..

[45] Medrano H., Escalona J.M., Cifre J., Bota J., Flexas J. (2003). A ten-year study on the physiology of two Spanish grapevine cultivars under field conditions: Effects of water availability from leaf photosynthesis to grape yield and quality. Funct. Plant Biol..

[46] Costa J.M., Ortuño M.F., Lopes C.M., Chaves M.M. (2012). Grapevine varieties exhibiting differences in stomatal response to water deficit. Funct. Plant Biol..

[47] Romero P., Fernández-Fernández J.I., Gil-Muñoz R., Botia P. (2016). Vigour yield quality relationships in long-term deficit irrigated winegrapes grown under semiarid conditions. Theor. Exp. Plant Physiol..

[48] Kolyva F., Rhizopoulou S., Meletiou-Christou M.S., Stratakis E. (2021). Physiological characteristics of expanding and expanded leaves of *Vitis vinifera* L. cv. Assyrtiko in climate change conditions. Biol. Life Sci. Forum.

[49] Pagay V., Furlan T.S., Kidman C.M., Nagahatenna D. (2022). Long-term drought adaptation of unirrigated grapevines (*Vitis vinifera* L.). Theor. Exp. Plant Physiol..

[50] Pou A., Balda P., Cifre J., Ochogavia J.M., Ayestarán B., Guadalupe Z., Llompart M., Bota J., Martínez-Lapuente L. (2023). Influence of non-irrigation and seasonality on wine colour, phenolic composition and sensory quality of a grapevine (*Vitis vinifera* cv. Callet) in a Mediterranean climate. OENO One.

[51] Koufos G.C., Mavromatis T., Koundouras S., Fyllas N.M., Theocharis S., Jones G.V. (2022). Greek Wine Quality Assessment and Relationships with Climate: Trends, Future Projections and Uncertainties. Water.

[52] Xyrafis E.G., Gambetta G.A., Biniari K. (2023). A comparative study on training systems and vine density in Santorini Island: Physiological, microclimate, yield and quality attributes. OENO One.

[53] Gómez-del-Campo M., Ruiz C., Lissarrague J.R. (2002). Effect of water stress on leaf area development, photosynthesis, and productivity in Chardonnay and Airén grapevines. Am. J. Enol. Vitic..

[54] Briglia N., Montanaro G., Petrozza A., Summerer S., Cellini F., Nuzzo V. (2019). Drought phenotyping in *Vitis vinifera* using RGB and NIR imaging. Sci. Hortic..

[55] Roig-Oliver M., Nadal M., Clemente-Moreno M.J., Bota J., Flexas J. (2020). Cell wall components regulate photosynthesis and leaf water relations of *Vitis vinifera* cv. Grenache acclimated to contrasting environmental conditions. J. Plant Physiol..

[56] Zamorano D., Franck N., Pastenes C., Wallberg B., Garrido M., Silva H. (2021). Improved physiological performance in grapevine (*Vitis vinifera* L.) cv. Cabernet Sauvignon facing recurrent drought stress. Aust. J. Grape Wine Res..

[57] Pagay V., Zufferey V., Lakso A.N. (2016). The influence of water stress on grapevine (*Vitis vinifera* L.) shoots in a cool, humid climate: Growth, gas exchange and hydraulics. Funct. Plant Biol..

[58] Jarvis A.J., Davies W.J. (1998). The coupled response of stomatal conductance to photosynthesis and transpiration. J. Exp. Bot..

[59] Gómez-Espinoza O., González-Ramírez D., Méndez-Gómez J., Guillén-Watson R., Medaglia-Mata A., Bravo L.A. (2021). Calcium oxalate crystals in leaves of the extremophile plant *Colobanthus quitensis* (Kunth) Bartl. (Caryophyllaceae). Plants.

[60] Botelho R.V., Roberti R., Tessarin P., Garcia-Mina J.M., Rombolà A.D. (2016). Physiological responses of grapevines to biodynamic management. Renew. Agric. Food Syst..

[61] Düring H. (1987). Stomatal responses to alterations of soil and air humidity in grapevines. Vitis.

[62] Merli M.C., Gatti M., Galbignani M., Bernizzoni F., Magnanini E., Poni S. (2014). Water use efficiency in Sangiovese grapes (*Vitis vinifera* L.) subjected to water stress before veraison: Different levels of assessment lead to different conclusions. Funct. Plant Biol..

[63] Merli M.C., Gatti M., Galbignani M., Bernizzoni F., Magnanini E., Poni S. (2015). Comparison of whole-canopy water use efficiency and vine performance of cv. Sangiovese (*Vitis vinifera* L.) vines subjected to a post-veraison water deficit. Sci. Hortic..

[64] Bertamini M., Zulini L., Zorer R., Muthuchelian K., Nedunchezhian N. (2007). Photoinhibition of photosynthesis in water deficit leaves of grapevine (*Vitis vinifera* L.) plants. Photosynthetica.

[65] Flexas J., Baron M., Bota J., Ducruet J.M., Galle A., Galmes J., Medrano H. (2009). Photosynthesis limitations during water stress acclimation and recovery in the drought-adapted *Vitis* hybrid Richter-110 (*V. berlandieri* × *V. rupestris*). J. Exp. Bot..

[66] Ye Q., Wang H., Li H. (2022). Arbuscular mycorrhizal fungi improve growth, photosynthetic activity and chlorophyll fluorescence of *Vitis vinifera* L. cv. Ecolly under drought stress. Agronomy.

[67] Kartal C. (2016). Calcium oxalate crystals in some species of the tribe Cardueae (Asteraceae). Bot. Sci..

[68] Gaberščik A., Grašič M., Vogel-Mikuš K., Germ M., Golob A. (2020). Water shortage strongly alters formation of calcium oxalate druse crystals and leaf traits in *Fagopyrum esculentum*. Plants.

[69] McDowell N. (2011). Mechanisms linking drought, hydraulics, carbon metabolism and vegetation mortality. Plant Physiol..

[70] McDowell N., Sevanto S. (2010). The mechanisms of carbon starvation: How, when, or does it even occur at all?. New Phytol..

[71] McDowell N., Pockman W.T., Allen A.D., Breshears D.D., Cobb N., Kolb T., Plaut J., Sperry J., West A., Williams D.G. (2008). Mechanisms of plant survival during drought: Why do some plants survive while others succumb to drought?. New Phytol..

[72] Scheuermann R., Biehler K., Stuhlfauth T., Fock H.P. (1991). Simultaneous gas exchange and fluorescence measurements indicate differences in the response of sunflower, bean and maize to water stress. Photosynth. Res..

[73] Biniari K., Xenaki M., Daskalakis I., Rusjan D., Bouza D., Stavrakaki M. (2020). Polyphenolic compounds and antioxidants of skin and berry grapes of Greek *Vitis vinifera* cultivars in relation to climate conditions. Food Chem..

[74] Hellenic National Meteorological Service. http://www.emy.gr/emy.

[75] Carrara M., Catania P., Pipitone F., Vallone M., Piraino S., Salvia M., Paolino C. (2008). Temperature and relative humidity distribution inside a greenhouse using wireless sensors. Acta Hortic..

[76] Barraqué B., Karavitis C., Katsiardi P., Koundouri E. (2008). The range of existing circumstances in the WaterStrategyMan case studies. Coping with Water Deficiency: From Research to Policymaking with Examples from Southern Europe, the Mediterranean and Developing Countries.

[77] Reid K., Oki L. (2013). Irrigation and climate zone trials of perennial plants for sustainable landscapes. Acta Hortic..

[78] Mapfumo E., Aspinall D., Hancock T.W. (1994). Growth and development of roots of grapevine (*Vitis vinifera* L.) in relation to water uptake from soil. Ann. Bot..

[79] Rai P., Mishra Ρ.Μ. (2013). Effect of urban air pollution on epidermal traits of road side tree species, *Pongamia pinnata* (L.) Merr. IOSR-JESTFT.

[80] Image-Pro Plus. http://www.mediacy.com/imageproplus.

[81] Knipfer T., Bambach N., Hernandez M.I., Bartlett M.K., Sinclair G., Duong F., Kluepfel D.A., McElrone A.J. (2020). Predicting stomatal closure and turgor loss in woody plants using predawn and midday water potential. Plant Physiol..

[82] Strasser R.J., Tsimilli-Michael M., Srivastava A., Papageorgiou E., Govindjee G.C. (2004). Analysis of the chlorophyll a fluorescence transient. Chlorophyll a Fluorescence: A Signature of Photosynthesis. Advances in Photosynthesis and Respiration.

[83] Stirbet A., Govindjee (2011). On the relation between the Kautsky effect (chlorophyll a fluorescence induction) and Photosystem II: Basics and applications of the OJIP fluorescence transient. J. Photochem. Photobiol. B Biol..

[84] Stirbet A., Lazár D., Kromdijk J., Govindjee (2018). Chlorophyll a fluorescence induction: Can just a one-second measurement be used to quantify abiotic stress responses?. Photosynthetica.

